# Adsorption of Lufenuron 50-EC Pesticide from Aqueous Solution Using Oil Palm Shell-Derived Activated Carbon

**DOI:** 10.3390/ma17215389

**Published:** 2024-11-04

**Authors:** David Nuñez, Juan Barraza, Juan Guerrero, Luis Díaz, Ajay K. Dalai, Venu Babu Borugadda

**Affiliations:** 1 Chemical Engineering School, Ciudad Universitaria Meléndez, Universidad del Valle, Calle 13 # 100-00, Cali 25360, Colombia; nunez.david@correounivalle.edu.co (D.N.); juan.guerrero.perez@correounivalle.edu.co (J.G.); 2 Valledupar Research and Development Center (CIDVA), Fundación Universitaria del Área Andina, Transversal 22 Bis #4-105, Valledupar 200005, Colombia; ldiaz164@areandina.edu.co; 3 College of Engineering, University of Saskatchewan, 57 Campus Drive, Saskatoon, SK S7N 5A9, Canada; ajay.dalai@usask.ca

**Keywords:** oil palm shell, activated carbon, adsorption, Lufenuron 50-EC pesticide

## Abstract

The use of Lufenuron 50-EC pesticide in oil palm crops affects water quality and aquatic life. This study investigated the adsorption of Lufenuron 50-EC from an aqueous solution using activated carbon derived from oil palm shells (OPSs). Activated carbon (AC) was prepared through physical and chemical activation processes in carbon dioxide environments, using potassium hydroxide (KOH) as a chemical activating agent. The resulting AC was characterized using standard techniques. The most favorable operating parameters were physical activation at 900 °C for 2 h, achieving a BET surface area of 548 m^2^/g. For chemical activation, at 800 °C, 1 h, and an impregnation ratio (KOH/biochar) of 2:1 (*w*/*w*), a BET surface area of 90 m^2^/g was obtained, which was smaller than that achieved by physical activation. The use of KOH reduced the surface area but generated a high presence of functional groups on the AC surface, which is important for adsorption processes. The AC produced achieved high Lufenuron adsorption yields, reaching a maximum of 96.93%. AC produced at 900 °C with 2 h showed the best performance. Therefore, OPS is an excellent precursor for producing AC with favorable characteristics for pollutant adsorption in aqueous solutions, especially for the insecticide Lufenuron.

## 1. Introduction

Activated carbon is a carbonaceous material with unique chemical and physical properties, whose main characteristics are high porosity and extensive internal surface area. These properties have promoted the usage of AC as catalyst supports and as direct fuels, given its high calorific value (HHV). Nevertheless, the physicochemical characteristics of AC depend on the activation process employed and the nature of the precursor. AC has exhibited a dominant capacity for the adsorption of diluted molecules both in the liquid and gaseous phases [1,2]. Owing to the adsorptive nature of AC’s, they have been investigated for the removal of a wide variety of organic and inorganic contaminants present in liquid or gaseous solutions [3]. Primarily, there are two processes used to synthesize AC, known as physical activation and chemical activation [4].

These two activation processes differ in certain aspects, mainly in the implementation of a chemical reagent (such as ZnCl_2_, NaOH, KOH, H_3_PO_4,_ or Na_2_CO_3_) for the impregnation of the precursor material or biocarbon in the case of chemical activation, making two additional stages within the process necessary (washing and drying). Additionally, these processes vary in terms of heat treatment, activation time, and oxidizing gas used [5,6,7]. These parameters define the final physical and chemical properties of the AC [8]. The choice of one of these activation routes or processes will depend fundamentally on the application needs of the AC. In parallel to the above, it is worth mentioning that work is being conducted with alternative adsorbent materials, such as the use of zirconium phosphate nanocrystals confined in polystyrene resins, demonstrating an efficient removal of cesium from an aqueous matrix [9], proposing it as an excellent alternative to address water contamination emergencies due to nuclear reactor accidents.

For the determination of physiochemical, surface, and optical properties of AC, standard techniques such as proximate and ultimate analysis, methylene blue index, internal surface area or BET adsorption, zero-point charge, FTIR, XRD, RAMAN, SEM, and elemental composition are generally used [10,11]. In general, AC can be obtained from a wide variety of precursors, such as olive seed, corn cobs, soybean stalks [12], banana stalks [13], hemp fiber waste [14], coals [15], and oil palm shell [4]. Due to the variety of biomass utilization, it is acknowledged to have great potential to produce ACs competent for their application towards industrial purposes [16].

According to the National Federation of Oil Palm Growers [17], Colombia remains the main oil palm producer in Latin America and in fourth place worldwide. According to the reports, the country produced 1,747,377 tons of crude palm oil and 7,882,225 tons of Fresh Fruit Bunches (FFB) in 2021, generating considerable quantities of oil palm shells each year [18]. In the Cesar State, Colombia, the production of palm oil for the year 2020 reached 241,905 tons (15.52% of national production), as it has 23 palm-producing municipalities, 8 processing plants, 77,869 planted hectares, and 68,604 hectares in production [19]. This makes the Cesar state one of the main producers in the country, with a great attraction for the development of new techniques oriented to the use of the waste generated during oil palm processing. By-products derived from oil palm processing are a promising raw material source if they are made available in an environmentally friendly way, socially acceptable, and profitable manner [20]. One of these by-products is the oil palm shell, proposed as a potential precursor to produce activated carbon and its application in adsorption processes [21,22].

The adsorption of pesticides in activated carbons derived from different precursors is a subject that has been worked on more frequently. A study [23] prepared activated carbon from orange waste by chemical activation with H_3_PO_4_ using activation temperatures between 500 and 900 °C, residence times between 1 and 6 h, and a heating rate of 10 °C/min for carbamate pesticide adsorption, obtaining maximum adsorption capacities *(q_m_)* between 7.97 and 93.46 mg/g. Similar results [16] were obtained using synthesized activated carbon from oil palm shell by physical activation for the adsorption of the paraquat pesticide using activation temperatures between 750 and 900 °C and residence time between 1 and 3 h under a constant flow of H_2_O, obtaining a *q_m_* of 109.7 mg/g.

In this work, AC was prepared through physical and chemical activation to give added value to a residue OPS in Colombia for its promising application in the adsorption of the Lufenuron pesticide commonly used in oil palm crops. This research represents a pioneering effort to address water pollution caused by the pesticide Lufenuron, using activated carbon derived from oil palm shells, a biomass source with high availability. Unlike previous studies, this work demonstrates the efficacy of using KOH as an activating agent to enhance the adsorption capacity of AC for the removal of the pesticide Lufenuron. These innovations set this study apart, offering both a novel approach to pesticide mitigation and valuable insights into sustainable waste valorization for environmental remediation. For chemical activation, KOH was used as an activating agent during the biochar impregnation process. The AC produced was characterized by proximate and ultimate analysis, Fourier transform infrared spectroscopy (FTIR), Brunauer–Emmett–Teller surface area (BET), X-Ray diffraction (XRD), Raman spectroscopy, and scanning electron microscopy (SEM) to understand their adsorption capability related to the chemical environment on the surface of the AC. Additionally, an adsorption isotherm study was carried out by comparing with the Langmuir and Freundlich models. The high adsorption levels obtained on activated carbons can be attributed to a strong affinity between the surface functional groups of AC and the Lufenuron molecule.

## 2. Materials and Methods

### 2.1. Materials

**Figure 1:**
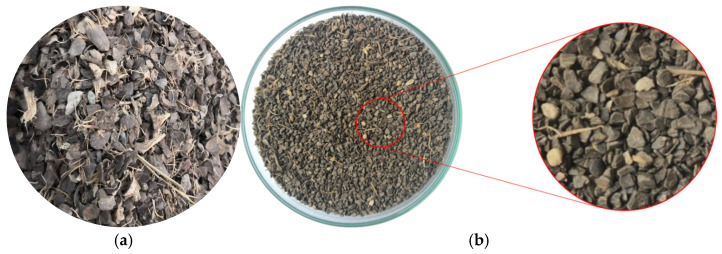
(**a**) OPS as received, (**b**) quartered representative sample.

A total of 33 kg of oil palm shells were sourced from one extractor company, a simplified stock company situated in Codazzi town, Cesar Colombia. These shells underwent a series of treatments, including drying, crushing via a fodder mill, and sieving to attain a particle size falling within the range of 0.8 to 2 mm, as seen in Figure 1. In the chemical activation process, it employed potassium hydroxide (KOH), a recognized chemical activator renowned for its ability to yield activated carbon with a high surface area [24].

The pesticide Lufenuron was procured from a pesticide store located in Cali, Colombia. Lufenuron is classified as a systemic insecticide within the Benzoylureas group. It functions as a growth regulator by suppressing chitin synthesis in insects. It falls under toxicological category III, signifying it has a slight level of toxicity with a molecular weight of 511.2 g/mol and a purity of 98%. This pesticide finds its primary application in oil palm crops, and specific characteristics of Lufenuron are shown in Table 1.

The pesticide Lufenuron poses a significant threat to aquatic biodiversity [25]. They exposed fish of a prominent species to varying concentrations of Lufenuron in water tanks over specific durations. The findings revealed adverse effects, including hepatocyte necrosis, in liver samples from the exposed fish. These outcomes not only jeopardize the health of aquatic ecosystems but also raise concerns regarding human health, particularly through the consumption of fish contaminated with this pesticide. A similar study [26] revealed that fish exposed to Lufenuron exhibited stress-related symptoms. Furthermore, Lufenuron presented various toxic effects in relation to the biological parameters of the exposed fish. The above generates a precedent to propose ideas aimed at preventing and addressing the contamination of aquatic biodiversity.

### 2.2. Preparation of Activated Carbon

The activation of the precursor material was carried out by physical and chemical processes. To obtain the biochar for both activation processes, 80 g of feedstocks (OPS) were pyrolyzed under the same operating parameters in a system of four stainless steel tubular reactors placed in a furnace. The reactor contents were subjected to a flow of purified nitrogen (N_2_) with a constant flow of 1061 cm^3^/min (purge time 10 min). The initial furnace temperature was 25 °C; the pyrolysis temperature was 700 °C; the heating rate was 10 °C/min; and the residence time was 30 min.

#### 2.2.1. Physical Process

**Figure 2:**
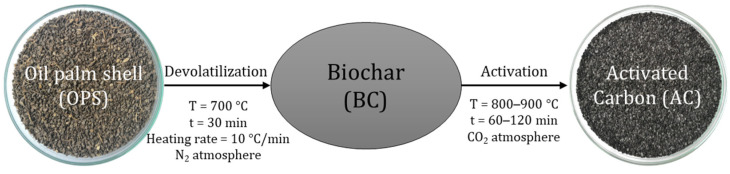
Physical activation process.

Devolatilization and activation were performed in one step. The obtained biochar at a temperature of 700 °C was physically activated at temperatures between 800 and 900 °C, residence times between 1 and 2 h, and a constant flow of carbon dioxide (CO_2_) of 201 cm^3^/min (Figure 2). Activation temperature and residence time were taken as independent variables, the choice of which was based on previous research [4]. A rotatable compound central experimental design was chosen with 2^2^ factorial treatments with 4 experimental runs and 1 central point with 3 replicates.

#### 2.2.2. Chemical Process

**Figure 3:**
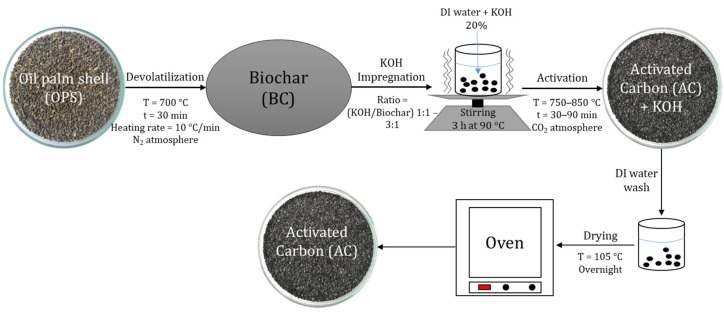
Chemical activation process.

In the chemical activation process, devolatilization and activation were performed in two steps. The biochar obtained from the devolatilization process was impregnated with a 20% *w*/*w* potassium hydroxide (KOH) solution with KOH/biochar ratios of 1/1 and 3/1. Each mixture of biochar and KOH solution was stirred for 3 h at a temperature of 90 °C to facilitate the impregnation process. Subsequently, the impregnated biochar was inserted in a system of four stainless steel tubular reactors and placed in a furnace under a constant flow of carbon dioxide (CO_2_) of 201 cm^3^/min. The activation temperatures ranged between 750 and 850 °C and residence times between 0.5 and 1.5 h (Figure 3). The activated carbon produced was washed with distilled water until reaching a neutral pH. Activation temperature, residence time, and impregnation ratio were taken as independent variables [24]. A rotatable compound central experimental design was chosen with 2^3^ factorial treatments with 8 experimental runs and 1 central point with 3 replicates.

AC yield from each run was also measured according to Equation (1).

AC yield (% *w*/*w*) = (W_2_/W_1_) × 100, (1)

where W_2_ is the final weight of the AC synthesized through physical and chemical activation, and W_1_ is the initial weight of the OPS.

### 2.3. Characterization of Precursor and AC Samples

Proximate and ultimate analysis of the precursor and AC samples was carried out according to ASTM (D3302/D3302M-19, D7582-15, D3172-13, D5865/D5865M-19, D4239-18e1 Method A, and D 5373-21 Method A, D3176-15, respectively), and the results were expressed in terms of volatile matter, fixed carbon, ash, HHV, and H, C, N, S, and O content on a dry basis (db). The functional groups on the surface of the samples and the precursor were analyzed by Fourier transform infrared spectroscopy (FT/IR—4100 type A) (Jasco Incorporated, Easton, MD, USA) (press KBr discs and the sample disc were scanned in a range from 4000 to 450 cm^−1^ and AC/KBr ratio of 1:100 was used). The textural properties of the samples were determined by implementing the equipment 3500 3Flex; (Micromeritics Instrument Corporation, Norcross, GA, USA) surface area (S_BET_), pore volume (V_T_), and pore size (S_P_) were determined by BET method and the Barret–Joyner–Halenda (BJH) model. Adsorption–desorption isotherms of nitrogen gas were obtained at 77 K, and degassing was carried out at a temperature of 300 °C for a period of 12 h. XRD technique was implemented to analyze the X-Ray diffractogram of the samples. To perform the XRD analysis, the Rigaku Ultima IV equipment (Rigaku Holdings Corporation, The Woodlands, TX, USA) was used, and the powder XRD data were collected in the two-theta range of 5–90°. InVia Raman Microscope (Renishaw plc, Mississauga, Canada) was used to detect the crystal structure, degree of disorder, defects, and graphitization in the activated carbons, and a JEOL JSM-6490LV SEM (JEOL BRASIL Instrumentos Cientificos Ltd., Sao Paulo, Brazil) was used to identify the physical morphology of the surface of the samples.

### 2.4. Batch Adsorption Process

The adsorption process was carried out in a shaker at an agitation speed of 160 rpm. Five experimental points were prepared for each activated carbon (0.05, 0.15, 0.25, 0.35, and 0.45 g) mixed with a solution of distilled water/Lufenuron with constant concentration (10 ppm). The AC and the 50 mL Lufenuron solution were placed in 50 mL glass containers and then taken to the shaker with a stirring ratio of 170 rpm at room temperature for 20 h. The pH of the solutions for the physically activated carbons was 7, and in the case of the chemically activated ones, it was 8 due to the high alkalinity of the KOH used as an impregnation reagent; once the adsorption process was conducted, the samples were analyzed by UV–visible spectrophotometry (UV–VIS Jasco V-730) at 219.6 nm wavelength to determine equilibrium concentrations.

Removal rate R (%) and the amount of adsorption at equilibrium q_e_ (mg/g) were determined by Equations (2) and (3), respectively.

(2) $$R=\frac{C_{0}-C_{e}}{C_{0}},$$

(3) $$q_{e}=\frac{\left(C_{0}-C_{e}\right)V}{m},$$

where C_0_ (mg/L) is the initial concentration of Lufenuron, C_e_ (mg/L) is the equilibrium concentration, V is the solution volume, and m is the AC mass.

## 3. Results and Discussion

### 3.1. Mass Yield of Activated Carbons

Table 2 shows the mass yields (*w*/*w*, % ash-free dry basis) of the activated carbons obtained through the physical activation process. The nomenclature used for sample identification is as follows: For example, sample AC-800-1 represents an activated carbon obtained through physical activation at 800 °C with a 1-h residence time. The standard deviation of the AC yields obtained by a physical process with respect to its mean (24.42%) was 1.57%.

As seen in Table 2, physically activated carbons showed a decreasing trend in mass yields as the activation temperature and residence time increased. Similar results were reported in previous studies [27]. As expected, it was found that the highest performance (26.4% *w*/*w*) was obtained at the activation temperature of 800 °C and 1 h of residence time, while the lower yield (21.8% *w*/*w*) was obtained at the most extreme operating conditions, activation temperature of 900 °C and 2 h residence time. This behavior may be attributed to increasing temperatures causing the release of volatile components in the precursor materials, reducing the overall mass and, consequently, reducing the yield of activated carbon. Also, elevated temperatures can influence the pore structure of the activated carbon, affecting its specific surface area and porosity, which can result in lower yields. The interaction of residence time (t) with temperature (T) is also a crucial factor. Longer activation times at higher temperatures may result in more extensive conversion and a decrease in the mass yield of the ACs.

Moreover, the activated carbons produced through physical activation displayed mass yields in the range of 21.8 to 26.4% *w*/*w*. This indicates that within the specified operating range, significant changes in mass yield occurred because of variations in activation temperature and residence time. The mass yields of AC, produced through physical activation, were lower compared to other carbonaceous residues, which exhibit higher yields on the order of 32% *w*/*w* [28].

Table 3 presents the mass yields of activated carbons obtained through the chemical activation process. The nomenclature used for sample identification is as follows: For example, the sample AC-750-0.5-3:1 represents an AC obtained through chemical activation at 750 °C, with 0.5 h of residence time and a KOH/biochar impregnation ratio of 3/1. The standard deviation of the AC yields obtained by chemical process with respect to its mean (26.57%) was 2.66%.

As observed in Table 3, the chemical AC showed mass yields ranging from 18.1 to 26% *w*/*w*. Just like in the physical activation process, these results demonstrate significant variations in mass yield with changes in activation temperature and residence time. It is worth noting that the mass yields of chemically activated carbons were lower when compared to other carbonaceous materials like apple pulp and olive stone, which were chemically activated with phosphoric acid (H_3_PO_4_) and achieved mass yields in the range of 32–38% *w*/*w* and 28.8–39.5% *w*/*w*, respectively [10,29]. It is important to highlight that the yields of AC depend on various factors, including the inherent characteristics of the precursor, the activation method, operating conditions, and the chemical agent used in the impregnation process.

The impregnation ratio (KOH/Biochar) in Table 3 played a significant role. Different impregnation ratios affected the chemical activation process. A higher ratio may lead to more extensive chemical reactions and a subsequent increase in AC yield. Additionally, this can also be influenced by the presence of KOH remaining on the surface of the ACs.

In general, physical activation (results in Table 2) produces higher AC yield percentages compared to chemical activation (results in Table 3). As can be seen, lower activation temperatures were used in the chemical process compared to the physical process. This temperature difference could lead to different chemical reactions during activation, which, in turn, affected performance.

### 3.2. Adsorption Yield of Lufenuron

The results corresponding to the adsorption yields of the pesticide Lufenuron obtained from the produced AC are shown in Table 4. The initial concentration of Lufenuron in the solution was 10 mg/L.

As seen in Table 4, in general, the AC produced in this study obtained satisfactory adsorption performances regardless of the activation method used, except for samples AC-800-1 (physical activation) and AC-750-0.5-3:1 (chemical activation) whose adsorption yields were below 65%. The highest yield obtained is attributed to the AC produced by physical activation at the temperature of 900 °C and residence time of 2 h, reaching a notable 96.93% removal of the pesticide from the Lufenuron solution.

Taking as reference the highest adsorption yields obtained during the process, two ACs were selected for each activation method to carry out a global physical-chemical characterization. The selected samples were AC-800-2 and AC-900-2 obtained for physical activation, while AC-750-1.5-3:1 and AC-800-1-2:1 for chemical activation.

### 3.3. Characterization of the Selected Activated Carbon

#### 3.3.1. Proximate and Ultimate Analysis of OPS and the ACs

The results of the proximate and ultimate analysis of the selected ACs obtained through physical activation (AC-800-2, AC-900-2) and chemical activation (AC-750-1.5-3:1, AC-800-1-2:1) on a dry basis, are showed in Table 5.

As expected, it was found that in general, the original OPS, compared to the AC samples, showed to have a higher content of volatile matter, lower content of fixed carbon, ash and lower high-heating value (HHV). These results are because of decreasing in the volatile matter of the original OPS, when it is subjected to pyrolysis at high temperatures.

The results of the proximate analysis also revealed differences in the carbon content between the ACs produced through physical activation (AC-800-2 and AC-900-2) and those obtained for chemical activation (AC-750-1.5-3:1, AC-800-1-2:1). This is due to the chemical reaction between KOH and some carbon atoms that takes place during the chemical activation process to accelerate pore formation. These findings are consistently with previous research [16,30,31].

On the other hand, the ash content reported in the proximate analysis of the AC holds significant relevance as it directly influences the surface area and porosity, factors that, in turn, affect adsorption capacity. A low ash content in activated carbon enhances its adsorption capacity, as demonstrated by [32]. As observed in Table 5, overall, the ash contents of the ACs obtained through both activation processes were higher compared to those exhibited by the original OPS precursor. This can be attributed to the reduction in volatile matter during the activation process.

As expected, the ACs produced show a significant increase in C compared to its precursor OPS, which is consistent with the fixed carbon recorded in the proximate analysis and the increase in HHV. Additionally, a substantial reduction in H and O in the AC is evident, as these components are typically associated with volatile matter. Consequently, during the release of volatiles, there is a reduction in H and O in the AC. In contrast, there is a slight increase in N in response to its relationship with C content. On the other hand, sulfur content showed no change.

A high HHV value indicates that the material is suitable for use as a fuel source and qualifies it as a good precursor for producing AC with a good adsorption capacity [33]. The HHV values obtained in this work are shown in Table 5, and they are very similar to the results reported by other authors [34,35,36]. A study [37] prepared biochar by pyrolysis of Miscanthus giganteus, obtaining HHV values between 22.6 and 32.8 MJ/kg. This biochar was used to prepare activated carbon and subsequently applied it in the adsorption of the pesticide Isoproturon, they concluded that these HHV values are a good indicator for using a material as a source of fuel or as activated carbon. Similar results were obtained by [38], who synthesized AC using KOH as an activating agent without using carbonaceous solids as a catalyst support. It was reported HHV values between 32.4 and 36.2 MJ/kg, concluding that the application of CA-KOH as a catalyst support is a promising medium for enhancing the catalytic activity of improved biofuels.

**Figure 4:**
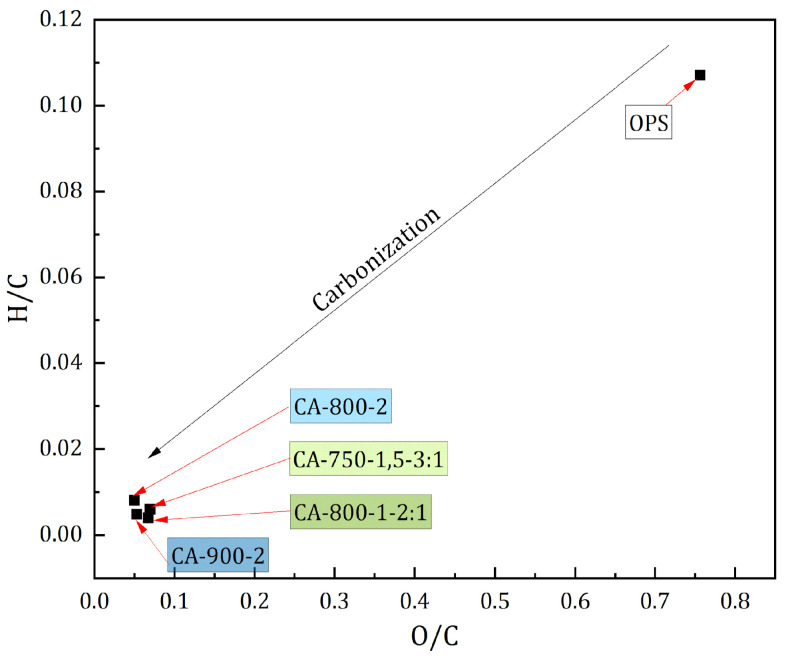
Van Krevelen diagram of OPS and selected ACs.

Figure 4 presents a Van Krevelen diagram depicting the hydrogen-to-carbon (H/C) atomic ratios versus oxygen-to-carbon (O/C) atomic ratios of the OPS, and for the selected ACs obtained physically and chemically.

The Van Krevelen diagram clearly illustrates the carbonization process during the activation of oil palm shell (OPS). This is evidenced by the changes in the H/C and O/C ratios, which show a significant reduction in hydrogen and oxygen content in the activated carbon (AC) compared to the OPS precursor. These shifts are indicative of the development of a highly carbon-rich structure with lower concentrations of hydrogen and oxygen, typical of carbonaceous materials.

#### 3.3.2. BET Surface Area, Volume and Pore Size of Selected ACs

**Figure 5:**
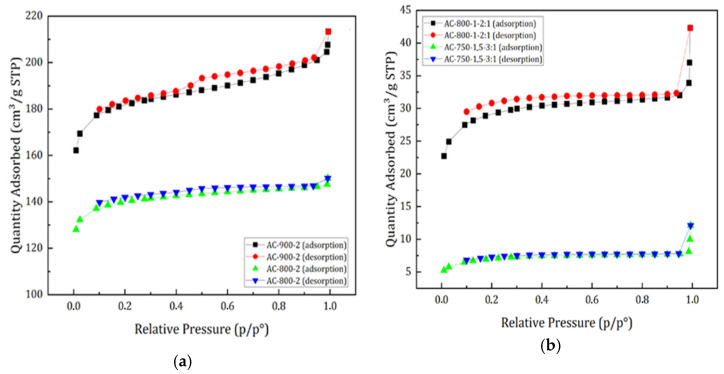
N2 adsorption–desorption isotherms at 77 K, physical activation (**a**), and chemical activation (**b**).

Evaluation of BET surface area, S_BET_ (m^2^/g), pore volume V_T_ (cm^3^/g), and pore size S_P_ distribution (nm) were determined by N_2_ adsorption–desorption isotherms at 77 K [39]. Figure 5 shows the results of the adsorbed amount of N_2_ for the ACs prepared by physical and chemical activation, respectively.

In Figure 5, it can be observed that the physically activated carbons reached nitrogen adsorption volumes in the range of 127–210 cm^3^/g, considerably higher than those obtained with the chemically activated carbons, which presented values in the range of 5–37 cm^3^/g. This can be attributed to the difference in surface areas developed by the ACs.

According to the classification of the International Union of Pure and Applied Chemistry (IUPAC), the isotherms obtained from the activated carbons produced in this study are of type I, with the exception of sample CA-900-2, which presented a type IV isotherm corresponding to mesoporous solids; they show a significant increase in adsorbed volume at relatively high pressures. These results agree with those reported by [23,30,40].

**Figure 6:**
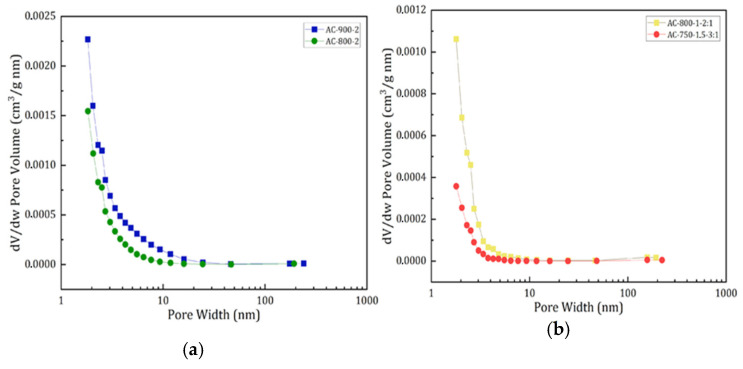
Pore size distribution of physical activation (**a**) and chemical activation (**b**).

Figure 6 presents the pore size distribution for activated carbons produced by physical (a) and chemical (b) activation.

It can be observed that both types of activation (physical and chemical) show a rapid decrease in pore volume at the smallest pore widths (1–10 nm), followed by a more gradual decrease up to 1000 nm, indicating a behavior typical of a pore size distribution. This finding suggests that the samples have a variety of pore sizes, with a gradual decrease in the number of larger pores, which is common in mesoporous materials. In addition, it is observed that CA by physical activation reaches higher pore frequencies as a function of their size than those presented in CA by chemical activation. This could be due to the differences in processing and activation conditions.

Additionally, it highlights the fact that in both activations, unimodal curves are presented, suggesting a more uniform pore size distribution, with greater presence in the size range of 2 to 50 nm. However, a significant amount of micropores is evident in the size range of 1.8 to 2 nm. This uniformity is a desirable characteristic, as it indicates a mesoporous structure on the surface of the AC. Mesoporous materials are particularly effective in adsorption applications due to their ability to accommodate molecules of different sizes. These results are comparable to those obtained in another study [41].

Table 6 presents the BET surface area and pore size data for biochar (BC) prepared by pyrolysis at 700 °C for 30 min with a value of 0.4 m^2^/g and 143.1 nm, respectively. The above results indicate that there is a need to increase the surface area and decrease the pores through thermal and chemical treatment processes.

Table 6 shows that the AC (AC-800-2 and AC-900-2) produced by physical activation presented the highest surface areas with values of 491 m^2^/g and 548 m^2^/g, total pore volume of 0.027 cm^3^/g and 0.080 cm^3^/g, as well as a mean pore size of 3.7 and 5.4 nm, respectively. In contrast, results for activated carbons (AC-750-1.5-3:1 and AC-800-1-2:1) obtained by chemical activation showed considerably smaller surface areas of 22 m^2^/g and 90 m^2^/g, with total pore volumes of 0.010 cm^3^/g and 0.027 cm^3^/g and average pore sizes of 7.3 and 6.4 nm, respectively. According to the above results, it is corroborated that all AC presented a mesoporous structure. Similar results in terms of BET surface area were recorded in the work [40,42], where palm kernels were used as a precursor to produce activated carbon with a coating of magnetite particles. In parallel, the similarity is reflected by the results obtained in other work [41], which used activated carbons derived from cane canary seed.

ACs produced by chemical activation showed a growth trend in the BET surface area with a decreasing impregnation ratio (KOH/BC) (% *w*/*w*). As seen in Table 6, sample AC-750-1.5-3:1 presented a value of 22 m^2^/g using an impregnation ratio of 3/1 compared to sample AC-800-1-2:1, which showed a value of 90 m^2^/g with an impregnation ratio of 2/1. These results could be due to the fact that the combination of the KOH activating agent with the subsequent treatment with CO_2_ contributes to a greater reaction of the biochar and, therefore, increases the diameter of the pores, which are larger and consequently decrease the surface area. However, it is pertinent to note that, even though these chemically activated carbons present low surface area, they are characterized by a greater presence of functional groups on their surface, which contributes to the increase in their adsorption capacity, as shown in the following sections.

#### 3.3.3. FTIR of Original OPS and Selected Activated Carbons

**Figure 7:**
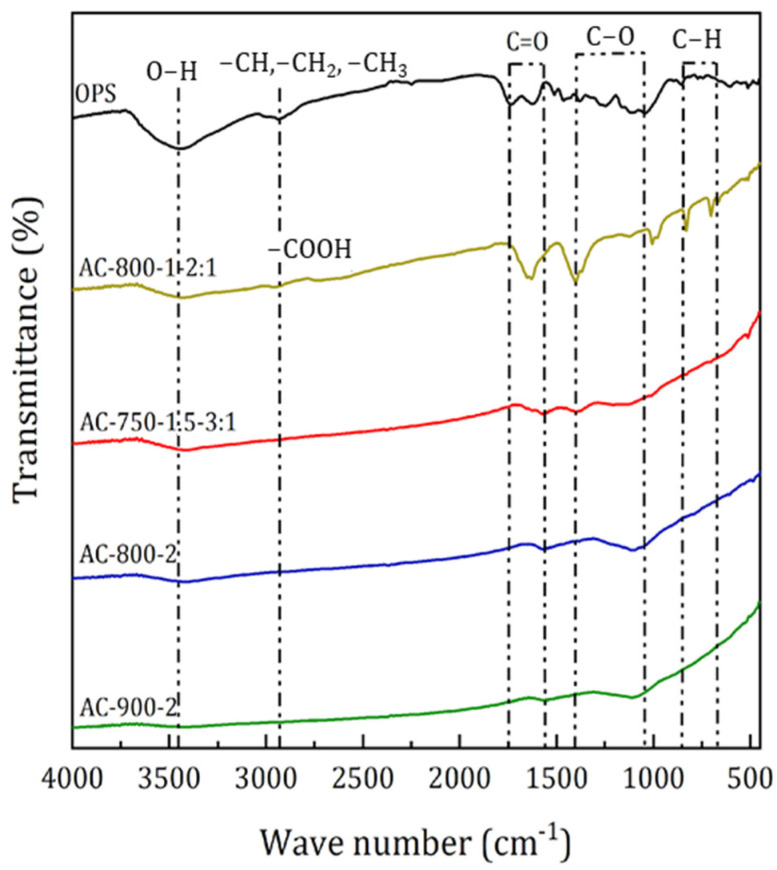
FTIR spectra of original OPS and selected ACs.

Figure 7 shows the FTIR spectra, while Table 7 presents the wavenumbers of the functional groups of both the OPS and the selected ACs obtained by physical and chemical activation.

In general, for OPS and Acs, it was found that the functional groups –OH, –CH, –CH_2_, –CH_3_, –COOH, C=O, –CO, and –CH were detected. The –OH functional group was detected in the wave range 3434–3423 cm^−1^ and is associated with the presence of water of hydration. It was identified that there was a decrease in the transmittance of the –OH group in AC (3423–3424 cm^−1^) compared to that presented by OPS (3434 cm^−1^). High hydrogen bond formation in OPS and low formation in AC may explain this behavior. These results agree with the fact that OPS naturally has a higher moisture content than AC.

The peak shown at wavelength 2931 cm^−1^ shows the presence of aliphatic groups (–CH, –CH_2_, –CH_3_) in the OPS, and the peaks at 2950–2890 cm^−1^ correspond to the appearance of carboxyl groups (–COOH) in the chemically activated carbons due to the use of KOH in the impregnation process, with the highest intensity in sample AC-750-1.5-3:1. The bands between 1738 and 1562 cm^−1^ denote the stretching vibration of the carbonyl group (C=O), while the bands between 1405 and 1400 cm^−1^ are attributed to the bending vibration of the C–O group.

Bands located at wavenumbers between 1122 and 1105 cm^−1^ indicate the presence of secondary alcohol by the C–O strain, and between 827 and 702 cm^−1^ indicate out-of-plane C–H bending in benzene derivatives. These results are comparable to those obtained in other works [12,43,44]. Also, results similar to those of this research were found in another study [45], which synthesized activated carbons from oil palm shells by physical activation with CO_2_ and chemical activation with KOH impregnation. The results showed that for the carbons activated chemically with KOH, there are functional groups present on the surface of alkali-type pyrones (cyclic ketone) and other keto derivatives of pyran, while in the case of the CA physically with CO_2_, there was the presence of aliphatic structures, phenols, and aromatic rings.

#### 3.3.4. X-Ray Diffraction of Original OPS, BC, and Selected Activated Carbons

**Figure 8:**
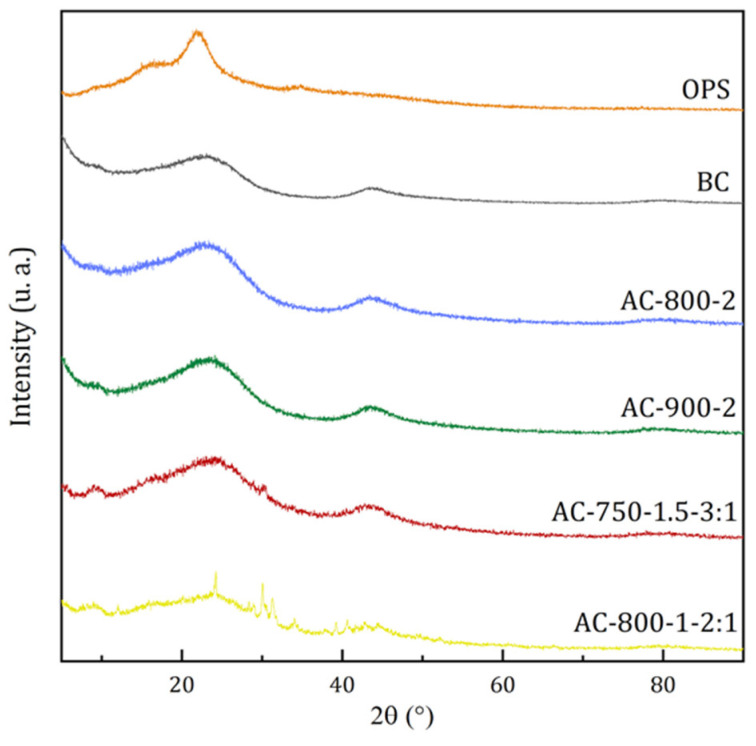
XRD analysis of OPS, BC, and ACs.

Figure 8 shows the XRD analysis of OPS, BC, and AC obtained at different activation conditions. The first two peaks at 2θ angles of 16 and 22° for the case of OPS are typical of the crystal structure of cellulose I_a_ (triclinic) and cellulose I_β_ (monoclinic) [36].

Once the OPS undergoes pyrolysis above 700 °C, a weakening in the degree of crystallinity of the cellulose or its degradation or transformation into other products is manifested, which is evident in the diffraction patterns obtained for BC and samples AC-750-1.5-3:1, AC-800-2, AC-800-1-2:1, and AC-900-2. Such patterns show a broadening of the diffraction peaks in the 20–30° and 40–45° ranges, corresponding to the characteristic graphite peaks and disordered graphitic plane (I_100_) due to the progressive stacking of graphene sheets and aromatic groups, indicative of the destruction of lignocelluloses in the carbon matrix, resulting in the formation of an amorphous carbon II-rich cellulose structure [46].

However, a weakening of these diffraction patterns is observed after chemical impregnation in samples AC-750-1.5-3:1 and AC-800-1-2:1, although no significant differences in diffraction patterns were detected. These results agree with another work [47]. Overall, this behavior is representative of an amorphous carbon crystal structure properly found in ACs, as reported in another work [22].

#### 3.3.5. Raman Spectroscopy of Selected Activated Carbons

**Figure 9:**
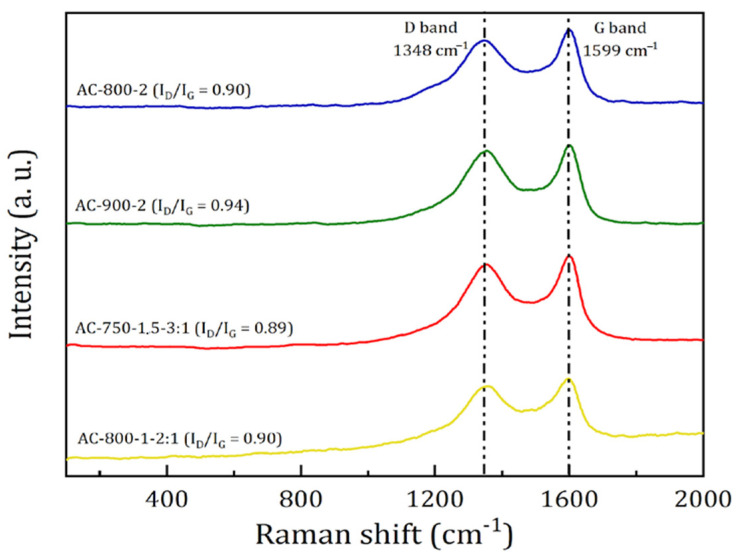
Raman spectra of selected ACs.

Raman spectroscopic analysis is a good technique to characterize the degree of graphitization of carbonaceous materials. Figure 9 shows the Raman spectra of the selected ACs obtained at different operating conditions.

As can be seen in Figure 9, two characteristic peaks were present around 1350 and 1560 cm^−1^, which are called D and G bands, respectively. The G band is related to the high presence of O–H functional groups on the surface of the activated carbon, which verifies what was found in the FT-IR analysis (Figure 7). These peaks are mainly attributed to the presence of sp^2^ carbon–carbon bonds in disordered microcrystalline domains of the ACs [48].

The degree of disorder in carbonized materials is assessed by the ratio between the intensities of the D and G bands (I_D_/I_G_). A higher ratio reflects a higher defect in the activated carbon and a higher degree of disorder in the atomic arrangement, i.e., they present a lower degree of graphitization [49,50]. It was found that the I_D_/I_G_ ratio increased with increasing pyrolysis temperature, from 0.89 to 0.94. These results mean that there existed a higher degree of defects and array disorder, as well as a lower degree of atomic graphitization in the ACs. This behavior could be a consequence of the gasification presented by the carbon, giving rise to greater defects within the carbon plane, as suggested by [51].

#### 3.3.6. SEM of Selected Activated Carbons

**Figure 10:**
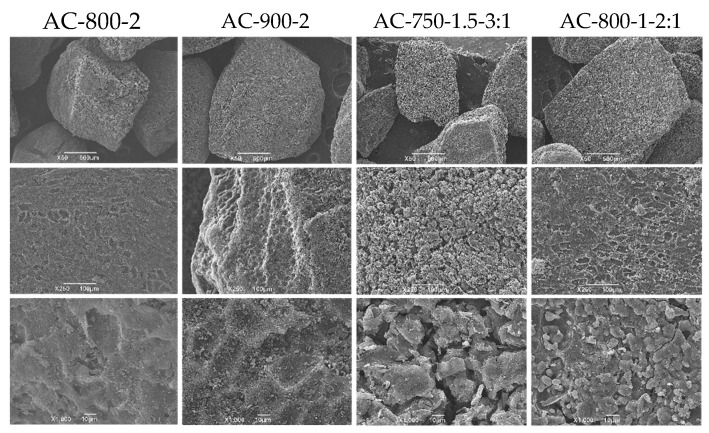
SEM micrographs of selected ACs.

Figure 10 shows the micrographs obtained by SEM corresponding to samples AC-800-2, AC-900-2, AC-750-1.5-3:1, and AC-800-1-2:1 using magnifications of 10, 100, and 500 microns.

In general, for all the ACs, the micrographs show cavities and pores with different dimensions and configurations in the activated carbons. These results are consistent with the work [52]. A work [24] established that the formation of these cavities and pores may be due to the release of volatile components and evaporation of potassium hydroxide (KOH), which previously occupied the active spaces on the surface of activated carbons during the chemical activation process.

As observed in the micrographs, there was a large presence of holes of different sizes, as well as cracks and crevices on the surface of the samples obtained by chemical activation (AC-750-1.5-3:1 and AC-800-1-2:1) compared to the carbons activated by the physical activation process (AC-800-2 and AC-900-2). These differences in the surface structures of the activated carbons obtained by physical and chemical activation are attributed to divergences in the nature of the activation processes, with the use of KOH as the most influential variable in producing these morphological differences. This is because KOH penetrates the carbon matrix and erodes the structure more aggressively. Larger cracks and holes are the result of expansion and fragmentation induced by the chemical interaction between KOH, carbon atoms, and CO_2_, which accelerates pore formation and breakdown of the carbon network during the activation process.

### 3.4. Adsorption Isotherms

Experimental tests were performed on samples AC-800-2, AC-900-2, AC-750-1.5-3:1, and AC-800-1-2:1 to determine the amount of Lufenuron pesticide adsorbed. Table 8 shows the maximum adsorption capacity for each AC.

The results presented in Table 8 show that the maximum adsorption capacities were in the range between 1011 and 1352 mg/g, with sample AC-900-2 reporting the highest maximum adsorption capacity of 1352 mg/g compared to the other ACs. This is due to the higher surface area presented (548 m^2^/g) compared to the other samples obtained by both physical and chemical activation.

It is important to note that, in general, for all adsorbents, pesticide adsorption percentages were higher than 90.2 (Table 4). This can be largely attributed to the hydroxyl (–OH), carbonyl (C=O), and, in the specific case of chemical activation, carboxyl (–COOH) functional groups on the surface of the activated carbons. A work [53] reported that hydroxyl groups in pesticides play an important role in adsorption processes due to their high affinity for adsorbents of carbonaceous nature. Another study [54] reported an adsorption percentage of pesticides similar to those found in this work, about 99%, using chemical activation with KOH under operating conditions of 400 °C activation temperature for 2 h, with pre-treatment of the precursor involving impregnation with sodium carbonate (Na_2_CO_3_) and pre-carbonization at 300 °C for 2 h. Other work [55] with similar results obtained a maximum percentage of pesticide removal from wastewater equal to 96.5%.

#### 3.4.1. Adsorption Isotherms for Selected AC Obtained by Physical Activation

**Figure 11:**
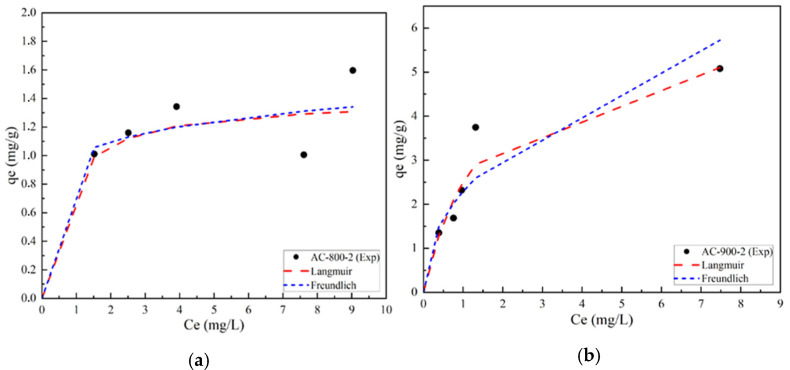
Adsorption isotherms of Lufenuron using physical AC (**a**) AC-800-2 and (**b**) AC-900-2.

Figure 11 shows the experimental equilibrium adsorption isotherms of Lufenuron pesticide for activated carbons AC-800-2 and AC-900-2, which are compared with the Langmuir and Freundlich mathematical models.

According to the classification of adsorption isotherms in water solutions, sample AC-800-2 shows an L-type isotherm. This behavior conforms precisely to the Langmuir isotherm. Figure 11a shows an initial behavior indicating a low adsorption capacity of the pesticide Lufenuron at low concentrations, which increases progressively with increasing concentration. Furthermore, a decrease in adsorption capacity is observed as the equilibrium concentration increases. This could be due to the saturation of adsorption sites on the adsorbent or other effects that limit adsorption at higher concentrations of the adsorbate.

In the case of sample AC-900-2 (Figure 11b), a behavior like that of sample AC-800-2 is observed: low adsorption capacity at low concentrations that gradually increase with increasing concentration, but in this case, higher adsorption capacities are observed. This can be attributed to the fact that sample AC-900-2 has a higher surface area and total pore volume, indicating the presence of a higher number of active sites available for the adsorption of the pesticide molecules. For both cases, AC-800-2 and AC-900-2, an asymptotic behavior is presented, indicating that a maximum adsorption capacity is reached. This type of isotherm fits precisely to the Langmuir model, which postulates that adsorption reaches its maximum when all active sites are covered by a monolayer of adsorbed molecules. According to this model, each active site has the capacity to host a single adsorbed molecule, which implies equal occupancy of all active sites and uniformity on the adsorbent surface. Furthermore, the Langmuir model assumes that the adsorption of a molecule on one site is not affected by the occupancy of adjacent sites [56,57].

#### 3.4.2. Adsorption Isotherms for Selected AC Obtained by Chemical Activation

**Figure 12:**
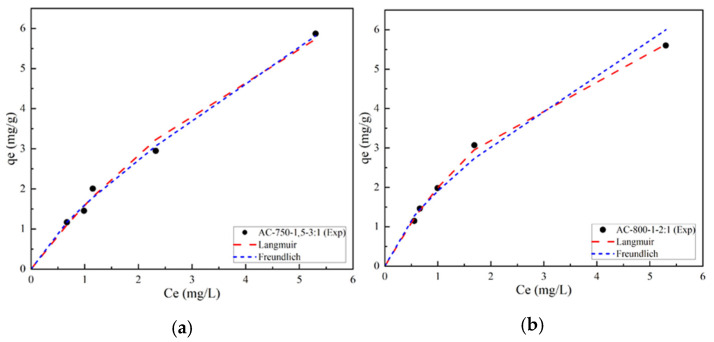
Adsorption isotherms of Lufenuron using chemical AC (**a**) AC-750-1.5-3:1 and (**b**) AC-800-1-2:1.

For sample AC-750-1.5-3:1 (Figure 12a), an adsorption profile is observed that follows a linear trend rather than converging asymptotically at high equilibrium concentrations. This suggests a proportional relationship between adsorption capacity and Lufenuron concentration, thus characterizing a behavior typical of C-type isotherms generally described by the Freundlich model. In contrast to the physically activated samples, where the adsorption capacity tends to stabilize, a steady increase in adsorption capacity is recorded in sample AC-750-1.5-3:1. This increase suggests that the active sites on the adsorbent surface have not become completely saturated, implying the availability of additional sites for the adsorption of more Lufenuron pesticide molecules.

In contrast, sample AC-800-1-2:1 (Figure 12b) shows how both models fit the experimental data well, but the Langmuir model fits slightly closer to the experimental points than the Freundlich model. Although the Pearson R^2^ coefficients for Langmuir and Freundlich are equal to 0.99 and 0.98 (Table 9), respectively, the Langmuir model has a slightly higher R^2^, suggesting a better fit to the experimental data. The Langmuir model assumes an adsorption monolayer with uniform occupancy of active sites, whereas the Freundlich model describes a multimonolayer adsorption with a heterogeneous distribution of active sites.

#### 3.4.3. Adsorption Parameters for the Selected Activated Carbons

With the experimental data obtained, regressions were performed using the Microsoft Excel 2013 solver tool v15.0 and corroborated with the OriginLab 2023 solver v10.0. Table 9 presents the values obtained for the adsorption equilibrium constants, adjusted with the Langmuir and Freundlich isotherms, with their respective Pearson R^2^ coefficients, for the analyzed samples AC-800-2, AC-900-2, AC-750-1.5-3:1, AC-800-1-2:1.

**Figure 13:**
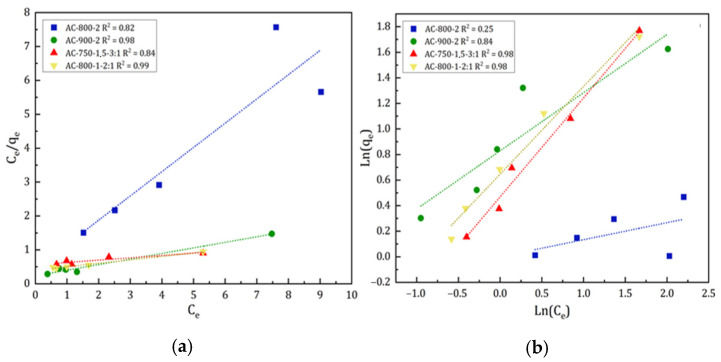
Linearized model of (**a**) Langmuir and (**b**) Freundlich.

The values of the Langmuir constants *q_m_* and *K_L_* were obtained by plotting *C_e_/q_e_* vs. *C_e_* (Figure 13a). In this representation, the slope corresponds to the *q_m_* value and the intercept to the *K_L_* value. On the other hand, the Freundlich constants *K_F_* and *n_F_* were determined from the slope and the intersection of the plot of *ln(q_e_)* vs. *ln(C_e_)* (Figure 13b).

The adsorption results of the pesticide Lufenuron on AC-900-2 and AC-800-1-2:1 activated carbons show that the experimental data fit more accurately to the Langmuir model, supported by the high values of Pearson’s correlation coefficient (0.98 and 0.99, respectively). These results are comparable to those found in other work [23], reporting fits above 0.98 for the adsorption of 6 different pesticides and adsorption capacities *q_m_* between 7.97 and 93.46. However, the activated carbon AC-900-2 showed an acceptable fit (0.84) to the Freundlich model. In the particular case of sample AC-800-1-2:1, a good fit (0.98) to the Freundlich model was also observed but fitting more accurately to the Langmuir model. On the other hand, the activated carbon AC-800-2, with an acceptable Pearson’s coefficient (0.82), fits experimentally better to the Langmuir model, considering that its experimental fit to the Freundlich model was considerably lower (0.25).

In contrast, the experimental data for activated carbons AC-750-1.5-3:1 showed a more accurate fit to the Freundlich isotherm, indicating the heterogeneity of the system and that this type of isotherm is not limited to the formation of a monolayer as activated carbons AC-800-2, AC-900-2, and AC-800-1-2:1 do. In summary, for AC-750-1.5-3:1 activated carbon, a saturation of the adsorption sites is not reached at high equilibrium concentrations, which means that it can continue to adsorb more molecules due to the heterogeneity of its adsorption sites and agrees with the results of another work [58].

Additionally, *R_L_* is a dimensionless equilibrium parameter that represents the essential characteristic factor of the Langmuir isotherm and is defined by Equation (4):

(4) $$R_{L}=\frac{1}{1+C_{0}K_{L}}\;$$

where *C*_0_ represents the initial concentration of Lufenuron in solution and *K_L_* the Langmuir constant signifying the adsorption rate. Values of *R_L_* = 0 indicate that the isotherm type is irreversible, 0 < *R_L_* < 1 favorable, *R_L_* = 1 linear, and *R_L_* > 1 unfavorable [24].

As shown in Table 9, the specific *R_L_* values obtained for samples AC-800-2, AC-900-2, AC-750-1.5-3:1, and AC-800-1-2:1 were 0.06, 0.10, 0.42, and 0.26, respectively. These results indicate that the Langmuir model was favorable for the adsorption of Lufenuron on the activated carbons produced experimentally in this study. However, despite this general trend, the sample AC-750-1.5-3:1 best fits the Freundlich model. This suggests that, although all the ACs exhibit favorable adsorption according to the Langmuir model, there is variation in adsorption among the different materials, with one of them being better suited to be described by the Freundlich model.

It is important to note that low values of the Langmuir *K_L_* constant are an indicator of high affinity between the adsorbate and the adsorbent [59]. Based on the *K_L_* values obtained, it can be assumed that the activated carbons developed in this study exhibit a marked affinity for the pesticide Lufenuron.

The maximum adsorption capacities (*q_m_*) obtained by fitting to the Langmuir model are presented in Table 9. It is observed that the adsorption capacities for the samples AC-750-1.5-3:1 were 14.54 mg/g, while those for AC-800-1-2:1 were 9.77 mg/g, which corresponds to the chemical activation process notably exceeding those achieved by AC-800-2 (1.4 mg/g) and AC-900-2 (6.09 mg/g), obtained through physical activation. This may be because the functional groups on the surface of chemically activated carbons interact more effectively with the adsorbate. This could explain why significant differences are observed between the maximum adsorption capacities of Lufenuron reported in Table 8 and the maximum adsorption capacities obtained by fitting to the Langmuir model (Table 9). Furthermore, it should be noted that the maximum adsorption capacities reported in Table 8 are experimental, while those reported in Table 9 are theoretical. The Langmuir model assumes monomolecular adsorption, while more complex interactions could occur in reality.

## 4. Conclusions

The nature of the activation process to obtain activated carbons affects their activation yields, mainly the activation temperature for physical activation and the residence time for chemical activation. The impregnation ratio had less relevance in the activation yield in chemically activated carbons but was of great relevance in the development of the BET surface area. The results obtained by proximate and elemental analyses qualify the oil palm shell as a potential precursor of activated carbon, and, in turn, the characteristics of the activated carbons obtained enhance its inherent capacity to adsorb contaminants in aqueous solutions.

The ACs produced exhibit a high fixed carbon content and a low ash content. The HHV values qualify it as an excellent fuel material. The use of KOH as a chemical reagent in the impregnation process considerably reduced the BET surface area. These results could be due to the fact that the combination of the KOH activating agent with the subsequent treatment with CO_2_ contributes to a greater reaction of the biochar and, therefore, increases the diameter of the pores, which are larger and consequently decrease the surface area. In turn, it generates a high presence and introduction of new functional groups such as carboxylic groups (–COOH). Using KOH as an impregnating agent significantly improves its adsorption capacity, which can be of great importance if adsorption processes are to be carried out.

The high adsorption levels (94–96.9%) obtained on activated carbons can be attributed to a strong affinity between the surface functional groups of AC and the Lufenuron molecule. In the context of activated carbon produced by physical activation, the high surface area means a higher number of sites available for these interactions. On the other hand, in the case of chemically activated carbons, where the surface area is considerably lower, the introduction of functional groups using KOH as an impregnating agent significantly enhances its adsorption capacity. In general, the activated carbons produced in this study exhibited competent adsorption yields, despite the evident variation in their surface areas. This suggests that adsorption capacity is not solely dependent on the surface area developed by each sample, but it may also be influenced by the nature of the surface functional groups and their interaction with Lufenuron.

## Figures and Tables

**Table 1:** Characteristics of Lufenuron.

Pesticide	Target Crop	Chemical Structure	Active Ingredient	Molecular Weight
Lufenuron 50-EC	Oil palm shell	(C_17_H_8_C_l2_F_8_N_2_O_3_) 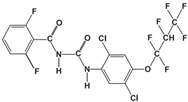	Lufenuron 50 g/L	511.2 g/mol

**Table 2:** AC yield is obtained by physical process on an ash-free dry basis.

Sample	Activation Temperature (°C)	Residence Time (h)	Mass Yield (wt. %)
AC-800-1	800	1.0	26.4
AC-800-2	800	2.0	25.5
AC-850-1.5	850	1.5	24.6
AC-900-1	900	1.0	23.8
AC-900-2	900	2.0	21.8

**Table 3:** AC yields obtained by chemical process, afdb.

Sample	Activation Temperature (°C)	Residence Time (h)	Impregnation Ratio (KOH/Biochar)	Mass Yield (wt. %)
AC-750-0.5-1:1	750	0.5	1:1	25.1
AC-750-0.5-3:1	750	0.5	3:1	26.3
AC-750-1.5-1:1	750	1.5	1:1	22.3
AC-750-1.5-3:1	750	1.5	3:1	22.9
AC-800-1-2:1	800	1.0	2:1	26.1
AC-850-0.5-1:1	850	0.5	1:1	24.7
AC-850-0.5-3:1	850	0.5	3:1	25.8
AC-850-1.5-1:1	850	1.5	1:1	18.1
AC-850-1.5-3:1	850	1.5	3:1	20.6

**Table 4:** Adsorption yields of Lufenuron from AC.

Activation Process	Sample	Activation Temperature (°C)	Residence Time (h)	Impregnation Ratio (KOH/Biochar)	Adsorption Yield (% *w*/*w*)
Physical	AC-800-1	800	1.0	N/A ^&^	57.16
	AC-800-2	800	2.0	N/A	90.15
	AC-850-1.5	850	1.5	N/A	90.11
	AC-900-1	900	1.0	N/A	90.10
	AC-900-2	900	2.0	N/A	96.93
Chemical	AC-750-0.5-1:1	750	0.5	1:1	89.05
	AC-750-0.5-3:1	750	0.5	3:1	64.93
	AC-750-1.5-1:1	750	1.5	1:1	93.42
	AC-750-1.5-3:1	750	1.5	3:1	94.01
	AC-800-1-2:1	800	1.0	2:1	94.87
	AC-850-0.5-1:1	850	0.5	1:1	93.25
	AC-850-0.5-3:1	850	0.5	3:1	87.86
	AC-850-1.5-1:1	850	1.5	1:1	92.94
	AC-850-1.5-3:1	850	1.5	3:1	92.18

N/A ^&^: Does Not apply.

**Table 5:** Analysis proximate and ultimate of OPS and selected AC, db.

Sample	Proximate (wt, %)			Ultimate (wt, %)					HHV (MJ/Kg)
	VM *	FC ^&^	Ash	C	H	N	O	S	
OPS	77.35	21.26	1.39	52.67	5.64	0.40	39.84	0.07	20.56
AC-800-2	8.71	85.73	5.56	88.71	0.72	0.51	4.43	0.07	30.33
AC-900-2	8.83	84.93	6.24	88.10	0.43	0.50	4.66	0.07	29.63
AC-750-1.5-3:1	16.58	71.57	11.86	81.37	0.49	0.56	5.65	0.07	28.41
AC-800-1-2:1	20.72	62.65	16.64	77.37	0.31	0.42	5.20	0.07	28.71

VM *: Volatile matter, db; FC ^&^: Fixed carbon, db.

**Table 6:** BET analysis of BC and selected ACs.

Sample	S_BET_ (m^2^/g)	V_T_ (cm^3^/g)	S_P_ (nm)
BC	0.4	0.01	143.1
AC-750-1.5-3:1	22.0	0.01	7.3
AC-800-2	421.0	0.03	3.7
AC-800-1-2:1	90.0	0.03	6.5
AC-900-2	548.0	0.08	5.4

**Table 7:** Functional groups of the OPS and selected ACs.

Sample	Wavelength (cm^−1^)					
	O–H	–CH, –CH_2_, –CH_3_	–COOH	C=O	C–O	C–H
OPS	3434	2931	N/A ^&^	1738–1628	1106	N/A^&^
AC-800-2	3423	N/A^&^	N/A	1562	1108	N/A
AC-900-2	3424	N/A	N/A	1562	1105	N/A
AC-750-1.5-3:1	3424	N/A	2950	1562	1400–1120	827–702
AC-800-1-2:1	3424	N/A	2950–2890	1637	1405–1122	827–702

N/A ^&^: Does Not apply.

**Table 8:** Lufenuron maximum adsorption capacity for ACs.

Sample	Maximum Adsorption Capacity (mg/g)
AC-800-2	1011
AC-900-2	1352
AC-750-1.5-3:1	1167
AC-800-1-2:1	1149

**Table 9:** Adsorption parameters for Lufenuron.

Model	Parameter	Sample			
		AC-800-2	AC-900-2	AC-750-1.5-3:1	AC-800-1-2:1
Langmuir	q_m_	1.40	6.09	14.54	9.77
	K_L_	1.61	0.69	0.12	0.26
	R^2^	0.82	0.98	0.84	0.99
	R_L_	0.06	0.10	0.42	0.26
Freundlich	K_F_	1.00	2.29	1.60	1.91
	1/n_F_	0.13	0.46	0.77	0.69
	R^2^	0.25	0.84	0.98	0.98

## Data Availability

The data presented in this study are available on request from the corresponding author because the data generated in this study are associated with a doctoral research project involving intellectual property rights. Sharing the data without control could compromise the authorship and the original work of the doctoral student.
